# Tailoring bismuth borate glasses by incorporating PbO/GeO_2_ for protection against nuclear radiation

**DOI:** 10.1038/s41598-021-87256-1

**Published:** 2021-04-08

**Authors:** Ashok Kumar, Anisha Jain, M. I. Sayyed, Farah Laariedh, K. A. Mahmoud, Jamel Nebhen, Mayeen Uddin Khandaker, M. R. I. Faruque

**Affiliations:** 1 University College, Benra, Dhuri, Punjab 148024 India; 2 Department of Physics, Punjabi University, Patiala, Punjab 147002 India; 3 Department of Physics, Faculty of Science, Isra University, Amman, 11622 Jordan; 4 Department of Nuclear Medicine Research, Institute for Research and Medical Consultations (IRMC), Imam Abdulrahman Bin Faisal University (IAU), PO. Box 1982, Dammam, 31441 Saudi Arabia; 5 Department of Physics, Faculty of Science, University of Tabuk, Tabuk, 47512 Saudi Arabia; 6 Tribology & Materials for Industry Laboratory, 69500 Bron, France; 7 Ural Federal University, St. Mira, 19, Yekaterinburg, Russia 620002; 8 Nuclear Material Authority, Maadi, Cairo, 530 Egypt; 9 College of Computer Science and Engineering, Prince Sattam Bin Abdulaziz University, PO.Box: 151, Alkharj, 11942 Saudi Arabia; 10 Center for Applied Physics and Radiation Technologies, School of Engineering and Technology, Sunway University, 47500 Bandar Sunway, Selangor Darul Ehsan Malaysia; 11 Space Science Centre (ANGKASA), Universiti Kebangsaan Malaysia (UKM), 43600 Bangi, Selangor Malaysia

**Keywords:** Materials science, Mathematics and computing, Physics

## Abstract

Nuclear radiation shielding capabilities for a glass series 20Bi_2_O_3_ − xPbO − (80 − 2x)B_2_O_3_ − xGeO_2_ (where x = 5, 10, 20, and 30 mol%) have been investigated using the Phy-X/PSD software and Monte Carlo N-Particle transport code. The mass attenuation coefficients (μ_m_) of selected samples have been estimated through XCOM dependent Phy-X/PSD program and MCNP-5 code in the photon-energy range 0.015–15 MeV. So obtained μ_m_ values are used to calculate other γ-ray shielding parameters such as half-value layer (HVL), mean-free-path (MFP), etc. The calculated μ_m_ values were found to be 71.20 cm^2^/g, 76.03 cm^2^/g, 84.24 cm^2^/g, and 90.94 cm^2^/g for four glasses S_1_ to S_4_, respectively. The effective atomic number (Z_eff_)values vary between 69.87 and 17.11 for S_1_ or 75.66 and 29.11 for S_4_ over 0.05–15 MeV of photon-energy. Sample S4, which has a larger PbO/GeO_2_ of 30 mol% in the bismuth-borate glass, possesses the lowest MFP and HVL, providing higher radiation protection efficiency compared to all other combinations. It shows outperformance while compared the calculated parameters (HVL and MFP) with the commercial shielding glasses, different alloys, polymers, standard shielding concretes, and ceramics. Geometric Progression (G-P) was applied for evaluating the energy absorption and exposure buildup factors at energies 0.015–15 MeV with penetration depths up to 40 mfp. The buildup factors showed dependence on the MFP and photon-energy as well. The studied samples' neutron shielding behavior was also evaluated by calculating the fast neutron removal cross-section (Σ_R_), i.e. found to be 0.139 cm^−1^ for S_1_, 0.133 cm^−1^ for S_2_, 0.128 cm^−1^ for S_3,_ and 0.12 cm^−1^ for S_4_. The results reveal a great potential for using a glass composite sample S4 in radiation protection applications.

## Introduction

γ-Rays emitting radionuclides are found to be useful in many fields like industrial (to detect defects in metal casting), medical (to treat malignant and cancerous tumors), agriculture (to control the degree of ripeness and extend the shelf life of fruits and vegetables) and space applications, etc.^1–3^. γ-Rays are high-frequency electromagnetic radiation that easily transmit through a thick wall, which may produce greater occupational exposures in nuclear facilities if not shielded adequately. Using suitable shielding may ensure better safety of radiation workers against its harmful dosages. Consequently, many researchers have been paid a great effort to develop and design well-formed radiation shielding materials^4–6^.

In this view, a range of materials, including concrete, lead, oxide glass, etc., have been developed and used to shield radiation^1,2,4^. Concrete has been formed from a loosely compacted mass of small fragments or particles^4,7^. It has several limitations to be used as shielding in a nuclear reactor. Due to its non-transparent characteristics, it is not possible to observe/monitor the internal environment. Moreover, variation in water quantity in concrete often exhibits unwanted fluctuations in attenuation coefficients^8,9^. To overcome such limitations, several glass-based materials have been processed. Being transparent in visible light, their properties can be modified by changing the components and preparation methods^8,10,11^. As a result, a number of researchers studied the γ-rays attenuation parameters for a variety of glasses like bismuth borate glasses^12^, lead borate glasses^13^, lead fluoroborate glasses^14^, bismuth borosilicate glasses^15^, alkali borosilicate glasses^16^, barium borosilicate glasses^17^, calcium–strontium-borate glasses^18^, lead silicate glasses^19^ and lead/barium phosphate glasses^20^, etc. In these glasses^12–20^, B_2_O_3_ is a common component that gives glass-forming of a lower melting point with good thermal stability and transparency. Smaller B^3+^ ionic size provides high bond strength^3,11^. Heavier cations used in different dosages promote glasses of varied shielding capabilities. Radiation shielding of glass structures can be developed by adding some high-density materials like heavy metal oxides (HMO).

The glass structures made with HMO (Pb, Ba, and Bi) show characteristics like high refractive index, high infrared transparency, and high nonlinear optical susceptibility^21–23^, all of which are favorable for a material to be used as an effective γ-rays shielding. Heavy metal oxides PbO, Bi_2_O_3,_ BaO, etc. play a key role in enhancing an average product density. A required characteristic of a good radiation shielding material is its chemical homogeneity made over its high density^24–26^. Generally, PbO containing high-density glasses offer high optical nonlinearity. A reasonably high PbO solubility is required to make a suitable glass for γ-rays' attenuation^27^. Bi_2_O_3_ doped borate glasses find wide applications in various fields like fast optical switching, photonic devices, and infrared transmission components, with high refractive index, large optical susceptibility, large polarizability, and high optical basicity^28^. Boro-germanate glasses offer high solubility to dissolve heavy metals, extending resistance to moisture, low melting point, good transparency, and excellent thermal stability^29^.

The main goal of this work is to evaluate the γ-rays shielding parameters of glasses 0.2Bi_2_O_3_ − xPbO − (0.8 − 2x)B_2_O_3_ − xGeO_2_, x = 0.05, 0.1, 0.2 and 0.3 mol %, as given in Table 1. The densities of the present samples have been taken from Knoblochova et al.^30^. The μ_m_ values, as determined using the XCOM dependent Phy-X/PSD program and MCNP computer code, were used to calculate the other shielding parameters like linear attenuation coefficient (µ), electron density, mean-free-path (MFP), half-value layer (HVL), and radiation protection efficiency (RPE). The exposure buildup factor (EBF) and energy absorption buildup factor (EABF) were evaluated using the geometric progression (GP) fitting method within a 0.015–10 MeV energy range. The interaction of the neutrons with the present glasses is studied in terms of fast neutron removal cross-sections.

**Table 1:** Chemical compositions and densities of studied samples.

Sample	Mole wight (mol%) of ingradients				Weight of elements (wt%)					Density (g/cm ^3^)
	Bi_2_O_3_	PbO	B_2_O_3_	GeO_2_	Bi	O	Pb	B	Ge	
S_1_	20	5	70	5	52.80	28.80	06.54	09.56	02.29	5.85
S_2_	20	10	60	10	49.83	25.75	12.35	07.73	04.33	6.01
S_3_	20	20	40	20	44.80	20.58	22.21	04.63	07.78	6.64
S_4_	20	30	20	30	40.69	16.35	30.25	02.10	10.60	7.02

## Theoretical approach on attenuating γ-rays

The MFP is defined as an average distance λ (in cm) traveled by the photons before being absorbed in a particular material. It has been calculated as^31–33^, MFP = μ^−1^, where μ (cm^−1^) denotes a linear attenuation coefficient of the medium. When a narrow beam of radiation of initial intensity I_0_ moves through a specific medium of thickness t, the number of photons (I) that can transmit the medium is given by the Lambert–Beer law^34–36^, I = I_0_e^−μt^. Also, the mixture rule is a suitable relation used to determine μ_m_ (or µ/ρ, where ρ is the material density) for an absorber^27,28^, µ_m_ = ∑ω_i_ (µ_m_)_i_. The HVL used to describe the material thickness diminishes the intensity I to be 0.5 I_0_^13^, HVL = 0.693 µ^−1^. The μ_m_ quantities helps in evaluating the total molecular cross-section (σ_t,m_)^13,36^, σ_t,m_ = µ_m_(M/N_A_), N_A_ is the Avogadros’ number. The σ_t,m_ used to calculate the average atomic cross-section (σ_t,a_)^12,36^, σ_t,a_ = σ_t,m_ (∑n_j_)^−1^. The fractional abundance f_i_, atomic mass A_i_, and atomic weight Z_i_ were used to calculate the average electronic cross-section (σ_t,el_), where σ_t,el_ = (N_A_)^−1^∑f_i_ A_i_ (µ_m_) (Z_i_)^−1^. The calculated quantities σ_t,a_ and σ_t,el_ utilized to calculate Z_eff_, Zeff = σ_t,a_ (σ_t,el_)^−1^. The radiation protection efficiency (RPE) of an attenuator is determined in a relation, RPE = (1 − e^−µt^) × 100.

The equivalent atomic number (Z_eq_) is interpolated by matching the ratio, R = (μ_m_)_comp_/(μ_m_)_total_. Beside, the Z_eq_, geometric progression (G-P) fitting parameters (b, c, a, X_k_ and d), (EABF), and (EBF) were calculated using the Phy-X/PSD program^37^.

On the other hand, a (∑_R_) represents the probability of a neutron undergoing certain reaction per unit length of moving through a certain medium, which can be calculated using the mass removal cross-section (∑_ER_) and fractional abundance ω for *i*^th^ constituent, ∑_R_ (cm^−1^) = ∑ω_i_ (∑_ER_)_i_.

## Simulations of shielding parameters

**Figure 1:**
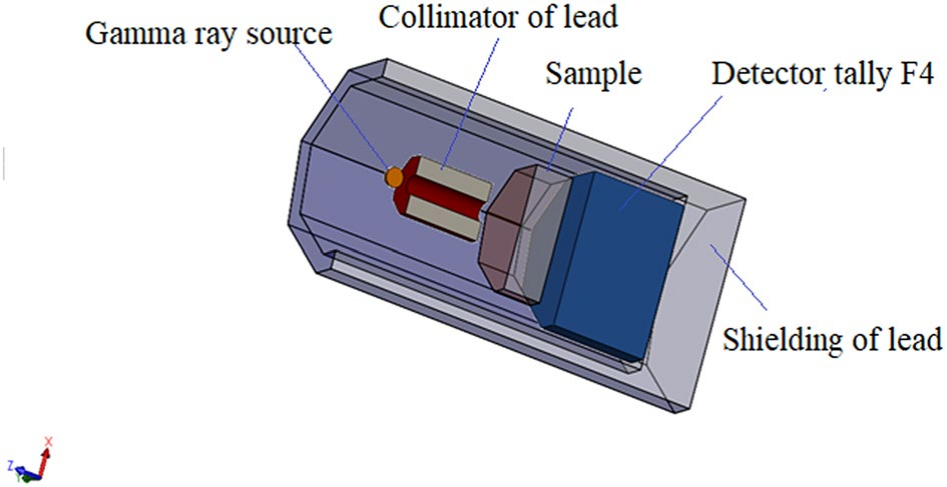
Schematic geometrical set-up for MCNP simulations.

The shielding parameters have been obtained using the user-friendly online Photon Shielding and Dosimetry (Phys-X/PSD) software. Several articles recently reported shielding properties against γ-rays, X-rays, and neutrons using simulation codes such as Geant, Fluka, and MCNP ^38–40^. Previously mentioned codes were used as alternative methods for the experimental measurements. The shielding parameters were evaluated using MCNP-5 code for the glasses 20 Bi_2_O_3_ − xPbO − (80 − 2x)B_2_O_3_ − xGeO_2_, with x = 5, 10, 20, and 30 mol %. As presented in Fig. 1, the simulation processes started with creating an input file, containing all information required to introduce the shielding material (density and chemical composition), γ-rays source (energy and its distribution), detector, and the geometry (cell and surface cards). A disk γ-source with a diameter of 2 cm and thickness of 0.5 cm was placed inside a lead collimator. The NPS card is set to stop running the simulation after 10^6^ particle. The sample was placed at mid-distance between the collimator and γ-rays detector, so that the γ-rays transmit via the sample and transmited part is directed to the detector. The simulation process aims to estimate the average track length (ATL) of γ-photons; thus, Tally (F4) was used. MCNP-5 is a helpful code supported by continuous-energy nuclear and atomic data libraries. The cross-section data sources used in the MCNP-5 nuclear database are ENDF/B-VI.8, ACTI, ENDL, ACTI, and T-16 files^41^.

## Results and discussion

**Figure 2:**
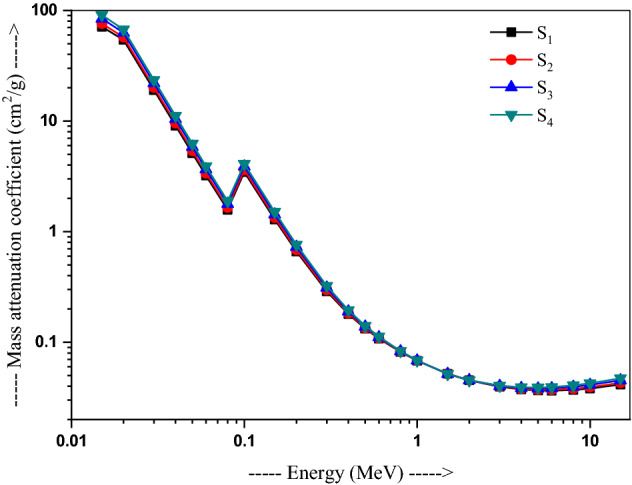
Variation of mass attenuation coefficient with energy in 20Bi_2_O_3_ − xPbO − (80 − 2x)B_2_O_3_ − xGeO_2_ (x = 5, 10, 20, and 30 mol%) glasses.

The μ_m_ values of the glasses simulated utilizing MCNP-5 code and calculated using the Phy-X/PSD in the energy range 0.015–15 MeV, as presented in Table 2. Both the μ_m_ values (MCNP-5 and Phy-X/PSD) are found to be in good agreement. Their variations with incident photon energies for all the glasses are displayed in Fig. 2. The μ_m_ values for sample S_1_ (71.20–1.288 cm^2^/g), S_2_ (76.03–1.34 cm^2^/g), S_3_ (84.24–1.43 cm^2^/g) and S_4_ (90.94–1.50 cm^2^/g) decrease sharply up to 15 keV, with a maximum over 71.20–90.94 cm^2^/g.

**Table 2:** Mass attenuation coefficients for the present glasses.

Energy (MeV)	Mass attenuation coefficient (cm^2^ g^−1^)											
	S_1_			S_2_			S_3_			S_4_		
	MCNP	Phy-X/PSD	Diff (%)	MCNP	Phy-X/PSD	Diff (%)	MCNP	Phy-X/PSD	Diff (%)	MCNP	Phy-X/PSD	Diff (%)
0.015	71.1273	71.2000	0.1023	75.9491	76.0400	0.1196	84.1213	84.2500	0.1530	90.7962	90.9500	0.1694
0.02	53.9431	54.1600	0.4022	57.1215	57.3500	0.3999	62.5140	62.7600	0.3936	66.9188	67.1800	0.3903
0.03	19.0596	19.0700	0.0544	20.1513	20.1600	0.0431	22.0034	22.0200	0.0754	23.5159	23.5300	0.0598
0.04	9.0488	9.0690	0.2237	9.5533	9.5740	0.2170	10.4101	10.4300	0.1915	11.1090	11.1300	0.1894
0.05	5.0815	5.1040	0.4437	5.3572	5.3810	0.4442	5.8249	5.8510	0.4483	6.2067	6.2340	0.4397
0.06	3.1842	3.2080	0.7477	3.3519	3.3770	0.7502	3.6362	3.6630	0.7358	3.8686	3.8970	0.7337
0.08	1.5461	1.5740	1.8025	1.6219	1.6510	1.7950	1.7505	1.7820	1.8007	1.8555	1.8890	1.8043
0.15	1.3769	1.2880	6.4555	1.4341	1.3420	6.4250	1.5733	1.4330	8.9194	1.6087	1.5080	6.2585
0.3	0.3092	0.2891	6.5102	0.3176	0.2974	6.3684	0.3318	0.3115	6.1109	0.3435	0.3230	5.9775
0.4	0.1792	0.1796	0.2351	0.1829	0.1834	0.2790	0.1892	0.1898	0.3265	0.1944	0.1950	0.3130
0.5	0.1324	0.1327	0.2585	0.1343	0.1347	0.2948	0.1430	0.1381	3.4599	0.1451	0.1409	2.8795
0.6	0.1071	0.1077	0.5552	0.1082	0.1088	0.5222	0.1102	0.1108	0.5792	0.1154	0.1124	2.6157
0.8	0.0813	0.0817	0.4701	0.0817	0.0821	0.4828	0.0824	0.0828	0.5075	0.0829	0.0834	0.5242
1.5	0.0510	0.0519	1.8278	0.0509	0.0519	1.9002	0.0507	0.0518	2.0549	0.0506	0.0517	2.1780
2	0.0448	0.0453	1.2209	0.0448	0.0453	1.2858	0.0447	0.0454	1.3656	0.0447	0.0454	1.4383
3	0.0394	0.0397	0.7390	0.0396	0.0399	0.7634	0.0399	0.0402	0.8121	0.0402	0.0405	0.8426
4	0.0373	0.0375	0.4957	0.0377	0.0379	0.5083	0.0383	0.0386	0.5253	0.0389	0.0391	0.5417
5	0.0365	0.0367	0.3667	0.0371	0.0372	0.3617	0.0380	0.0382	0.3891	0.0388	0.0389	0.3862
6	0.0364	0.0365	0.3241	0.0371	0.0372	0.3077	0.0382	0.0383	0.3112	0.0392	0.0393	0.3190
8	0.0370	0.0371	0.2181	0.0379	0.0380	0.2200	0.0394	0.0395	0.2023	0.0407	0.0408	0.2120
10	0.0381	0.0382	0.1788	0.0392	0.0393	0.1723	0.0411	0.0411	0.1554	0.0426	0.0426	0.1576
15	0.0414	0.0415	0.1328	0.0429	0.0429	0.1124	0.0453	0.0453	0.1277	0.0472	0.0473	0.1119

**Figure 3:**
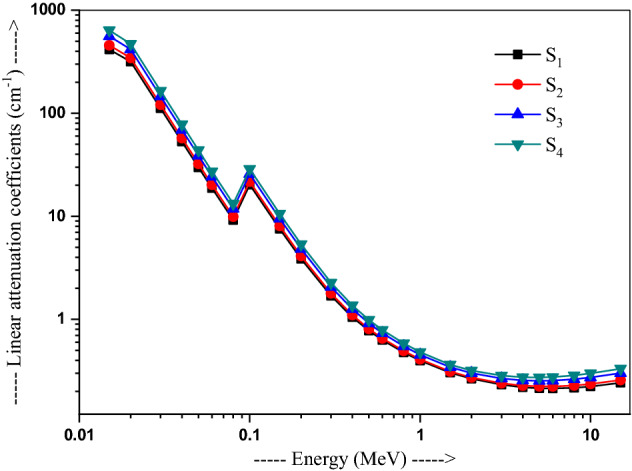
Variation of linear attenuation coefficient with energy in 20Bi_2_O_3_ − xPbO − (80 − 2x)B_2_O_3_ − xGeO_2_ (x = 5, 10, 20, and 30 mol%) glasses.

An abrupt change in μ_m_ is observed in the lower energy region in the Pb/Bi modified glasses, which have their K—absorption edges. The highest μ_m_ value is shown in S_4_ what is it required for a good shielding. In this energy region, the photoelectric process (PE) is the dominant process, which has the Z-dependence of Z^4–5^. The μ_m_ values for all samples (~ 0.08 cm^2^/g) are found to be nearly constant in the energy range 0.08 MeV on predominating Compton scattering, which varies linearly with Z, falling down on higher energies. The μ_m_ is found to increase slowly above 1 MeV on prevailing pair production process in this region, i.e., an order of Z^2^. It is found to be 0.037–0.041 cm^2^/g in S_1_, 0.038–0.043 cm^2^/g in S_2_, 0.039–0.045 cm^2^/g in S_3_, and 0.039–0.047 cm^2^/g in S_4_ in the energy range of 4–15 MeV. The linear attenuation coefficients can easily be obtained from the μ_m_ values, following a similar trend with the energy as presented in Fig. 3^42^. Table 2 shows a comparison of these values closely lying one another. The diff (%) calculated between the two programs is ≤ 5%.

**Figure 4:**
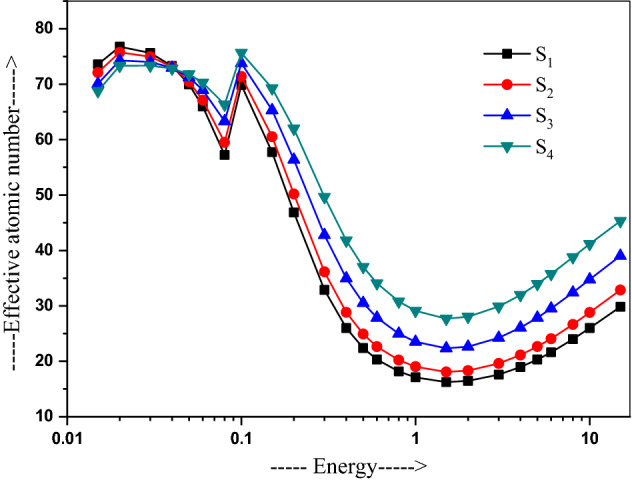
Variation of the effective atomic number with energy in 20Bi_2_O_3_ − xPbO − (80 − 2x)B_2_O_3_ − xGeO_2_ (x = 5, 10, 20, and 30 mol%) glasses.

Figure 4 plots the Z_eff_ changes with the energy in the different samples, varied over 69.87–17.11 for S_1_ and 75.66–29.11 for S_4_ in the energy range of 0.05–15 MeV. The Z_eff_ is found to decrease up to 1.5 MeV on dominance of the photoelectric absorption process in this region, which has Z-dependence of Z^4–5^. It arises sharply beyond 3 MeV, attributing to dominance of pair production process, which depends on Z^2^. At 15 MeV, it is found to be 29.83 for S_1_, 32.86 for S_2_ 39.01 for S_3_, and 45.30 for S_4_. A maximum value are used in S_4_ over S_1_ in a duly increased PbO dose. A low value 17.11–17.60 for S_1_ and 29.11–29.89 for S_4_ in a medium 1–3 MeV energy region is contributed by the dominant Compton scattering in this region, which has a linear Z-dependence responsible for duly increased Z_eff_ in the high-energy regions^43^.

**Figure 5:**
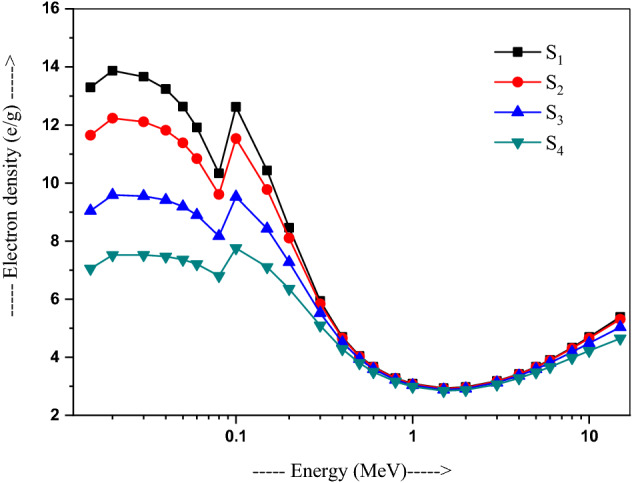
Variation of electron density with energy in 20Bi_2_O_3_ − xPbO − (80 − 2x)B_2_O_3_ − xGeO_2_ (x = 5, 10, 20, and 30 mol%) glasses.

The N_e_ values, calculated for present samples at different γ-rays energies using Eq. (9), are ploted with energy in Fig. 5. These are 1.33 × 10^24^ e/g (i.e. electrons/g) for S_1_ and 7.05 × 10^23^ e/g for S_4_ at 15 keV. The values of S_1_ (2.94 × 10^23^ e/g) and S_4_ (2.84 × 10^23^ e/g), with a minimum at 1.5 MeV, fall down sharply up to 1 MeV. A pretty smaller value is found for S_1_ of 3.09 × 10^23^–3.18 × 10^23^ e/g, while 2.99 × 10^23^–3.07 × 10^23^ e/g for S_4_ in the medium 1–3 MeV energy region, and then increases beyond 3 MeV. The Z_eff_ values at 15 MeV are 5.39 × 10^23^ and 4.65 × 10^23^ e/g for glass samples S_1_ and S_4_, respectively.

**Figure 6:**
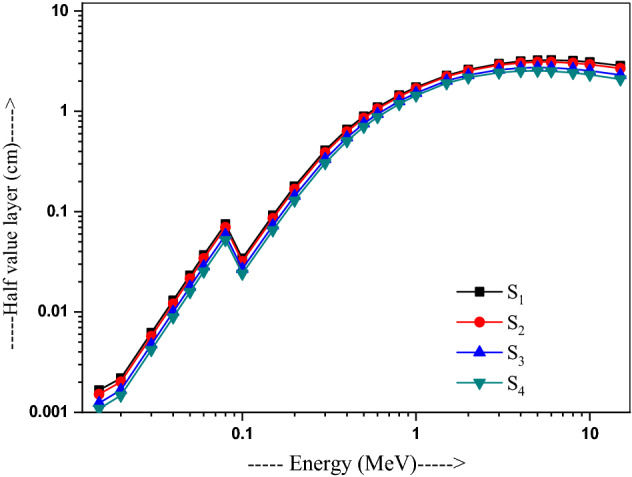
Variation of the half-value layer with energy in 20Bi_2_O_3_ − xPbO − (80 − 2x)B_2_O_3_ − xGeO_2_ (x = 5, 10, 20, and 30 mol%) glasses.

Figure 6 presents how HVL is varied with photon energy. It is significantly lower and stationary below 0.1 MeV and then arises sharply above 0.1 MeV, having the values 0.002 and 0.001 cm at 15 keV, while 0.034 and 0.024 cm at 0.1 MeV for S_1_ and S_4_, respectively. It becomes 3.24, 3.01, 2.73 and 2.51 cm at 6 MeV, while 2.857, 2.688, 2.303 and 2.088 cm at 15 MeV for S_1_, S_2_, S_3_ and S_4_, respectively. A minimum value shown in glass S_4_ thus characterizing it to be the best shielding material^42,43^ among all the four glasses studied here.

**Figure 7:**
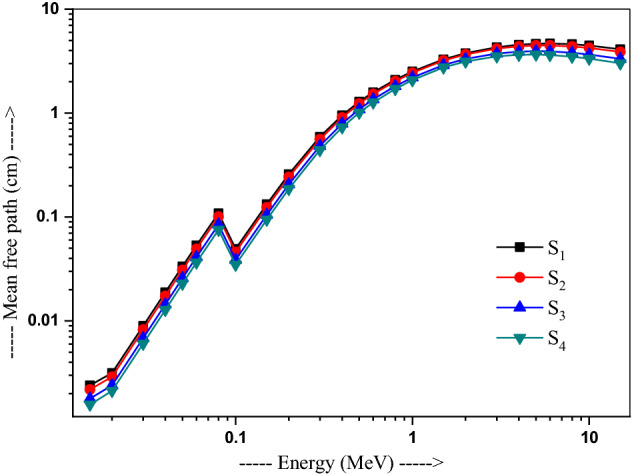
Variation of the mean free path with energy in 20Bi_2_O_3_ − xPbO − (80 − 2x)B_2_O_3_ − xGeO_2_ (x = 5, 10, 20, and 30 mol%) glasses.

The MFP values, as calculated from μ at several energies, vary from 0.015 to 15 MeV, as plotted in Fig. 7. Evidently, MFP varies with energy due to dominance of photons interactions. All the four S_1_–S_4_ glasses have a value 0.02 cm at 15 keV energy. A sharp peak is marked at 80 keV, showing 0.109, 0.101, 0.085 and 0.075 cm values (MFP) for S_1_, S_2_, S_3_, S_4_, respectively. Significantly lower (nearly steady) values stand below 0.1 MeV, namely, 0.04 cm, 0.04 cm, 0.03 cm and 0.03 cm at 0.1 MeV for the respective samples, following the dominance of photoelectric effect, and quickly arise over 0.3–6 MeV energy on Compton scattering dominates. Those become 4.681 cm, 4.474 cm, 3.928 cm, and 3.626 cm respectively at 6 MeV. Above 6 MeV, almost constant MFP prolongs on dominance of pair production, namely, 4.121 cm, 3.878 cm, 3.323 cm and 3.012 cm, respectively, at 15 MeV^42^. The shielding effectiveness is thus better in the lower energies, i.e. glass S_4_ is the best attenuator.

**Figure 8:**
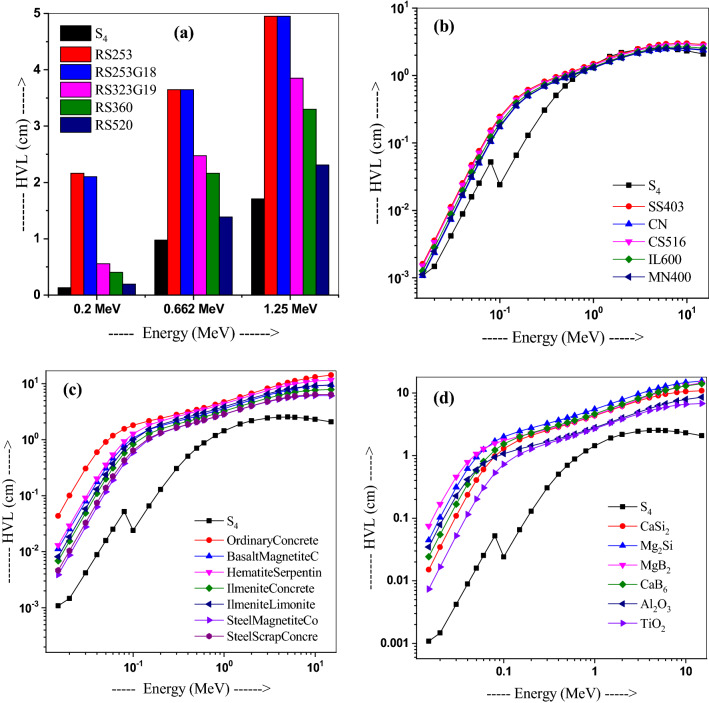
Comparison of HVL values of glass S4 with those in (**a**) commercial shielding glasses, (**b**) alloys, (**b**) alloys, (**c**) concretes and lead, and (**d**) ceramics materials.

**Figure 9:**
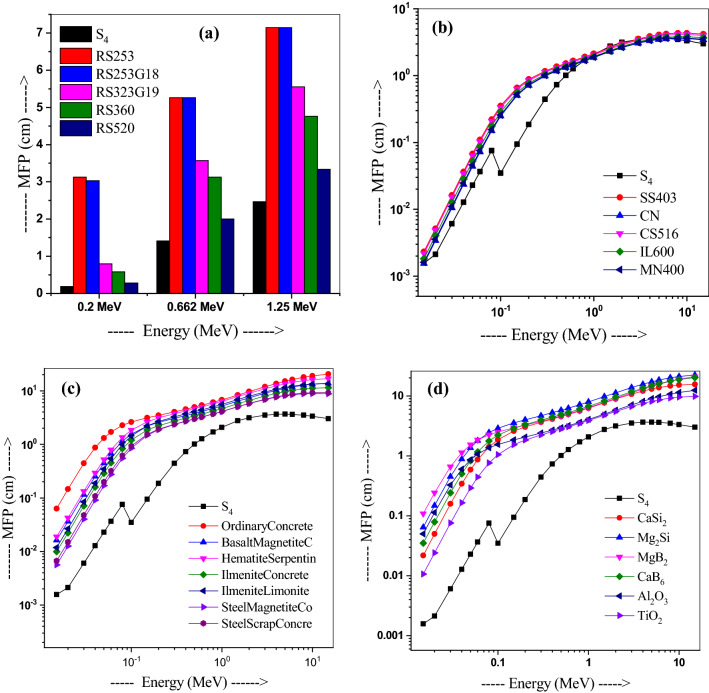
Comparison of MFP values of glass S4 with those in (**a**) commercial shielding glasses, (**b**) alloys, (**c**) concretes and lead, and (**d**) ceramics materials.

Further, the HVL and MFP of sample S_4_ have been compared to that of traditionally used shielding materials such as five types of glasses fabricated by SCHOTT AG, steels such as stainless steel-403 (SS403), cupero-nickel (CN), carbon steel-516 (CS516), inconel-600 (IL600), and monel-400 (MN400) alloys^44^, several types of concretes^45^ and ceramics such as CaSi_2_, Mg_2_Si, MgB_2_, CaB_6_, Al_2_O_3_, or TiO_2_, as shown in Figs. 8a–e and 9a–e respectively. Usefully, our glass S_4_ possesses better HVL and MFP values compared to that of traditionally used shielding materials. Values of both HVL and MFP for alloys are higher than the sample S_4_ values, except at 1.5 and 2 MeV (Figs. 8b and 9b). Moreover, Figs. 8c,d and 9c,d show lower HVL and MFP values of sample S4 as compared to alloys and concretes. Thus, S_4_ possess better shielding ability compared to the commercial glasses, concretes, alloys (excluding at 1.5 and 2 MeV energies), or ceramics.

**Figure 10:**
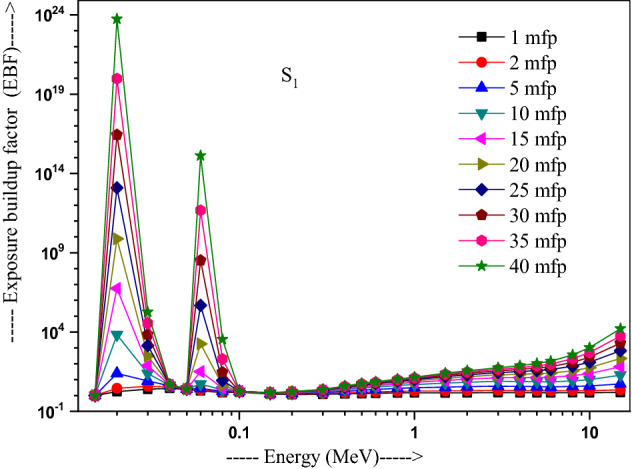
Variation of exposure buildup factor with energy for 20Bi_2_O_3_ − xPbO − (80 − 2x)B_2_O_3_ − xGeO_2_ (x = 5) glass (S_1_).

**Figure 11:**
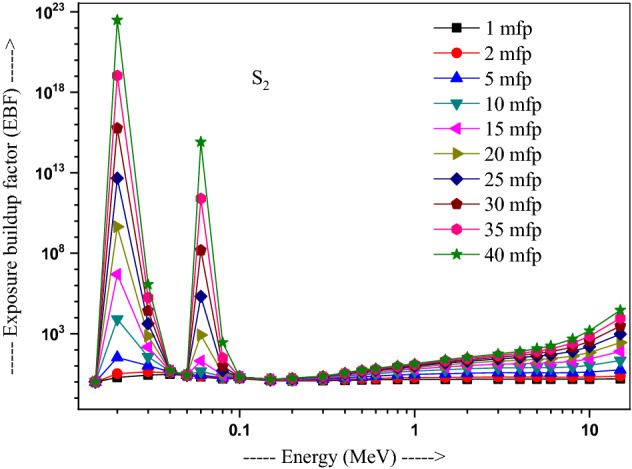
Variation of exposure buildup factor with energy for 20Bi_2_O_3_ − xPbO − (80 − 2x)B_2_O_3_ − xGeO_2_ (x = 10) glass (S_2_).

**Figure 12:**
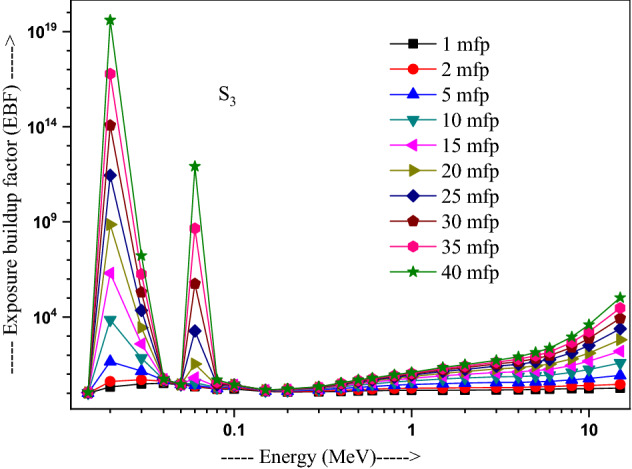
Variation of exposure buildup factor with energy for 20Bi_2_O_3_ − xPbO − (80 − 2x)B_2_O_3_ − xGeO_2_ (x = 20) glass (S_3_).

**Figure 13:**
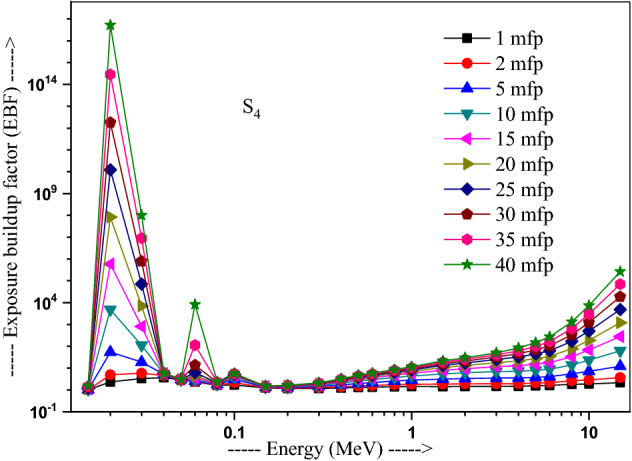
Variation of exposure buildup factor with energy for 20Bi_2_O_3_ − xPbO − (80 − 2x)B_2_O_3_ − xGeO_2_ (x = 30) glass (S_4_).

**Figure 14:**
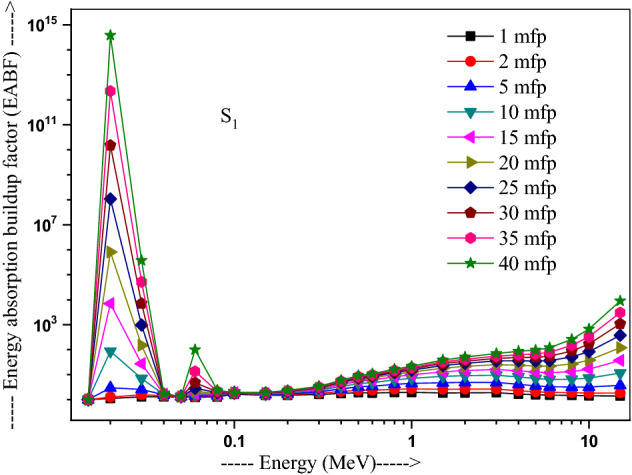
Variation of energy absorption buildup factor with energy for 20Bi_2_O_3_ − xPbO − (80 − 2x)B_2_O_3_ − xGeO_2_ (x = 5) glass (S_1_).

**Figure 15:**
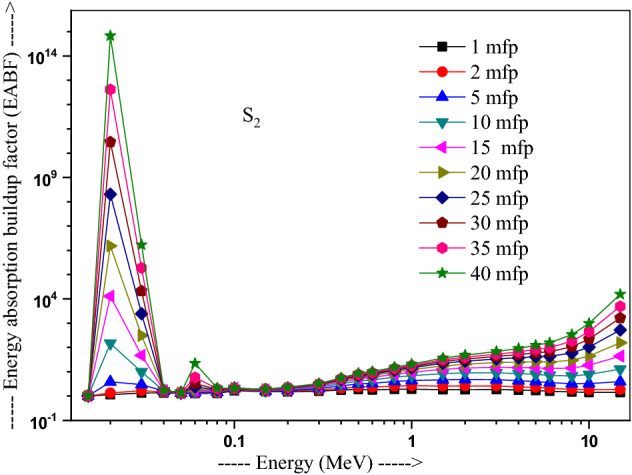
Variation of energy absorption buildup factor with energy for 20Bi_2_O_3_ − xPbO − (80 − 2x)B_2_O_3_ − xGeO_2_ (x = 10) glass (S_2_).

**Figure 16:**
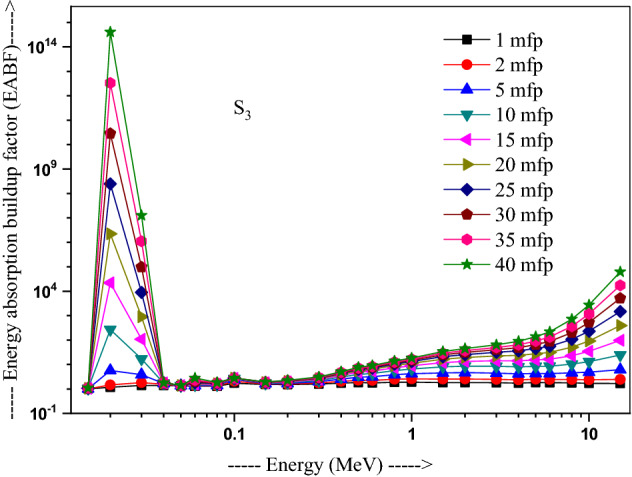
Variation of energy absorption buildup factor with energy for 20Bi_2_O_3_ − xPbO − (80 − 2x)B_2_O_3_ − xGeO_2_ (x = 20) glass (S_3_).

**Figure 17:**
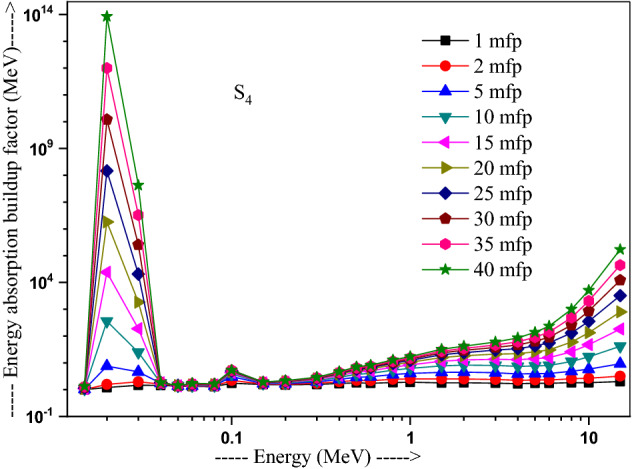
Variation of energy absorption buildup factor with energy for 20Bi_2_O_3_ − xPbO − (80 − 2x)B_2_O_3_ − xGeO_2_ (x = 30) glass (S_4_).

The values of other parameters studied for the present glasses are given in Table 3. The changes in EBF and EABF studied with energy at several penetration depths of 1, 2, 5, 10, 15, 20, 25, 30, 35 and 40 mfp are plotted in Figs. 10, 11, 12 and 13, 14, 15, 16 and 17 respectively. Both EBF and EABF of the studied samples possess low values in low and high-energy regions, but assume higher values in the moderate energy regions. At 0.015 and 0.15 MeV energies, EBF and EABF values are more dependent on sample contents and increase with decreasing Z_eq_ in these glasses. The Z_eq_ is maximum in S_4_, while minimum in S_1_. Both EBF and EABF in low-energies of 0.015–0.3 MeV are small and nearly equal to one for all penetration depths since the photons are totally absorbed/removed through photoelectric absorption in dominant interaction process up to 0.3 MeV. Those progressively increase with energy due to multiple Compton scattering (the degradation of photon energy), which dominates in the intermediate-energies (0.3–3 MeV). The EABF reduces in high-energy region (E > 3 MeV) in absorption behavior of the pair production process. After that, for gamma photon energies higher than 3 MeV, the buildup factors have high increase with increasing the incident energy. Also, EBF has a peak at 0.02 MeV in the K-absorption edge of high Z-elements present in these glasses^46^. The EBF values are the highest for 40 mfp and lowest for the penetration depth of 1 mfp due to the multiple scattering events for large penetration depths. Therefore, both EBF and EABF are increasing to reach a maximum for all S_1_, S_2_, S_3,_ and S_4_ samples for penetration depth at 40 mfp. But, the buildup factors are maximum/minimum for S_1_/S_4_ at penetration depths of 1, 2, 5, 10, 15, 20, 25, 30, 35 and 40 mfp for incident energies up to 3 MeV. By contrast, at E > 3 MeV, S_4_ has maximum EBF and S_1_ has the least EBF.

**Table 3:** Equivalent atomic numbers and G-P fitting parameters for EBF and EABF for sample S_4_.

Energy (MeV)	Z_eq_	G-P fitting parameters—EBF					G-P fitting parameters—EABF				
		a	B	c	d	X_k_	a	b	c	d	X_k_
1.50E−02	33.41	− 0.106	1.004	1.402	0.114	11.712	− 0.115	1.004	1.408	0.124	12.378
2.00E−02	40.01	0.120	2.353	1.775	− 0.150	12.970	0.075	1.198	1.807	− 0.074	17.415
3.00E−02	40.20	0.120	3.331	1.005	− 0.198	29.753	0.123	1.455	1.013	− 0.126	26.025
4.00E−02	40.47	0.103	3.651	0.323	− 0.039	22.476	0.116	1.444	0.327	− 0.069	23.443
5.00E−02	40.76	− 0.272	2.944	0.064	0.040	12.036	− 0.122	1.358	0.076	0.100	8.673
6.00E−02	41.02	1.048	2.325	0.027	− 0.147	17.265	0.764	1.314	0.043	− 0.188	14.886
8.00E−02	41.50	0.748	1.646	0.046	− 0.251	14.386	0.561	1.298	0.091	− 0.225	14.051
1.00E−01	69.94	0.095	1.707	0.624	− 0.060	17.221	0.100	1.733	0.603	− 0.063	17.125
1.50E−01	71.19	0.385	1.238	0.146	− 0.131	14.776	0.410	1.560	0.094	− 0.097	21.530
2.00E−01	71.86	0.323	1.156	0.277	− 0.181	13.781	0.602	1.514	0.088	− 0.295	13.912
3.00E−01	72.61	0.165	1.178	0.494	− 0.079	13.645	0.386	1.572	0.214	− 0.213	13.319
4.00E−01	73.04	0.121	1.242	0.607	− 0.064	14.148	0.289	1.696	0.334	− 0.182	13.716
5.00E−01	73.32	0.097	1.299	0.677	− 0.053	14.131	0.232	1.808	0.424	− 0.151	13.747
6.00E−01	73.51	0.079	1.343	0.726	− 0.043	13.693	0.165	1.727	0.541	− 0.105	13.584
8.00E−01	73.71	0.056	1.405	0.799	− 0.033	13.709	0.127	1.840	0.629	− 0.085	13.581
1.00E+00	73.80	0.042	1.438	0.854	− 0.028	13.343	0.105	1.876	0.693	− 0.076	13.530
1.50E+00	73.17	0.012	1.427	0.981	− 0.020	14.278	0.058	1.793	0.845	− 0.056	13.837
2.00E+00	71.22	0.005	1.446	1.020	− 0.020	13.334	0.064	1.796	0.848	− 0.069	13.422
3.00E+00	66.69	0.017	1.465	1.017	− 0.042	13.305	0.091	1.749	0.804	− 0.109	13.533
4.00E+00	63.56	0.033	1.469	0.986	− 0.057	13.740	0.104	1.659	0.787	− 0.122	13.869
5.00E+00	61.62	0.065	1.559	0.906	− 0.085	13.973	0.138	1.728	0.718	− 0.153	14.137
6.00E+00	60.44	0.072	1.606	0.900	− 0.090	14.175	0.140	1.726	0.725	− 0.153	14.285
8.00E+00	59.02	0.071	1.811	0.940	− 0.089	14.165	0.126	1.828	0.788	− 0.142	14.280
1.00E+01	58.25	0.035	1.895	1.103	− 0.058	14.041	0.085	1.831	0.939	− 0.109	14.075
1.50E+01	57.42	0.008	2.169	1.319	− 0.039	13.682	0.047	1.999	1.161	− 0.082	13.764

**Figure 18:**
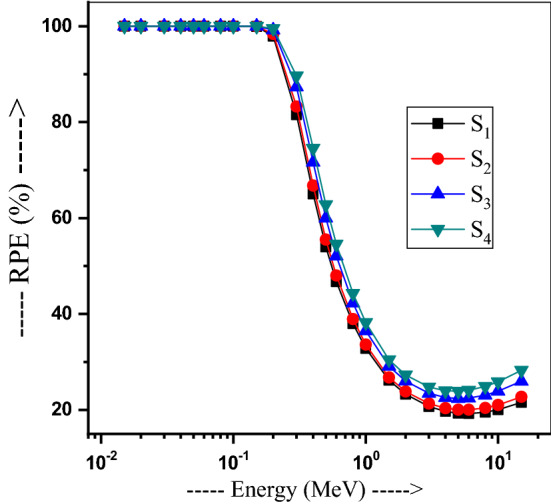
Variation of the radiation protection efficiency with energy for 20Bi_2_O_3_ − xPbO − (80 − 2x)B_2_O_3_ − xGeO_2_ glasses.

**Figure 19:**
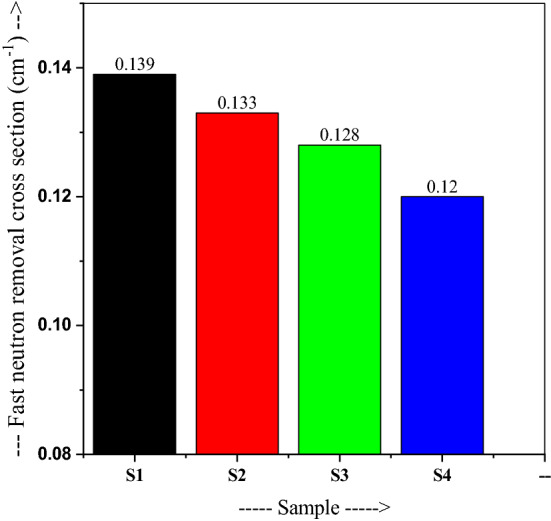
The fast neutron removal cross-section for 20Bi_2_O_3_ − xPbO − (80 − 2x)B_2_O_3_ − xGeO_2_ glasses.

Calculated RPE for S_1_-S_4_ glasses and their variation with energy is portrayed in Fig. 18, where RPE is stationary up to 0.1 MeV. This means the incident photons can totally penetrate at 0.1 MeV, then RPE sharply decreases over 0.1 to 3 MeV energies, showing 23.29, 23.58, 26.00, and 27.28% residual values in respective glasses, which stay constant up to 8 MeV. About 21% of incident photons penetrate at 15 MeV. Glasses S_1_ and S_4_ have 32.83% and 38.20% values at 1 MeV, respectively, in a due effect of PbO–GeO_2_ additives of suppressing the attenuation properties. Thus, sample S4 can shield better than the other glasses. A composite glass has the property of removing more neutrons if it owes high Z elements. Low-Z elements may also remove neutrons if one using a combination of high-Z elements with low-Z elements. As portrayed in Fig. 19, the Σ_R_ value varies as 0.139, 0.133, 0.128 and 0.12 cm^−1^ in the respective glasses. There is only a minor variation in this parameter. The amount of Z-elements like B and O may increase the neutron shielding capability in such glasses.

## Conclusions

Major γ-rays shielding parameters are studied over a multicomponent glass series, 20Bi_2_O_3_ − xPbO − (80 − 2x)B_2_O_3_ − xGeO_2_, with x-values varied in small steps up to 30 mol%, in precisely determining how sensitively the PbO/GeO_2_ additives tailor the features. The additives promptly facilitate proportional behavior for µ, Z_eff_ and RPE in the doped glasses on duly suppressed HVL and MFP values. A sample S_4_ is found to possess the highest value of the effective atomic number and the sample S_1_ is found to possess the lower values of effective atomic number among the selected glasses beyond 0.05 MeV, anticipating a synergic PbO role of improving the shielding capability in this series. The HVL and MFP of this sample S_4_ have been compared to those of traditionally used shielding glasses, such as SCHOTT AG glasses, steels, polymers, concretes, and ceramics. This specific glass S_4_ possesses suitably lowered HVL and MFP values (excluding at 1.5 and 2 MeV energies), over all these traditionally materials being used for this purpose. This work reveals a great potential of selected lead borate glasses to shield ionizing radiations in nuclear environment. The future scope of the presently selected glass series is to explore the structural and the mechanical properties.
